# Comparison of 4D Flow MRI to 2D Flow MRI in the pulmonary arteries in healthy volunteers and patients with pulmonary hypertension

**DOI:** 10.1371/journal.pone.0224121

**Published:** 2019-10-24

**Authors:** Malte Maria Sieren, Clara Berlin, Thekla Helene Oechtering, Peter Hunold, Daniel Drömann, Jörg Barkhausen, Alex Frydrychowicz

**Affiliations:** 1 Department of Radiology and Nuclear Medicine, University Hospital Schleswig-Holstein, Lübeck, Germany; 2 Department of Pneumology, University Hospital Schleswig-Holstein, Lübeck, Germany; McLean Hospital, UNITED STATES

## Abstract

**Purpose:**

4D and 2D phase-contrast MRI (2D Flow MRI, 4D Flow MRI, respectively) are increasingly being used to noninvasively assess pulmonary hypertension (PH). The goals of this study were i) to evaluate whether established quantitative parameters in 2D Flow MRI associated with pulmonary hypertension can be assessed using 4D Flow MRI; ii) to compare results from 4D Flow MRI on a digital broadband 3T MR system with data from clinically established MRI-techniques as well as conservation of mass analysis and phantom correction and iii) to elaborate on the added value of secondary flow patterns in detecting PH.

**Methods:**

11 patients with PH (4f, 63 ± 16y), 15 age-matched healthy volunteers (9f, 56 ± 11y), and 20 young healthy volunteers (13f, 23 ± 2y) were scanned on a 3T MR scanner (Philips Ingenia). Subjects were examined with a 4D Flow, a 2D Flow and a bSSFP sequence. For extrinsic comparison, quantitative parameters measured with 4D Flow MRI were compared to i) a static phantom, ii) 2D Flow acquisitions and iii) stroke volume derived from a bSSFP sequence. For intrinsic comparison conservation of mass-analysis was employed. Dedicated software was used to extract various flow, velocity, and anatomical parameters. Visualization of blood flow was performed to detect secondary flow patterns.

**Results:**

Overall, there was good agreement between all techniques, 4D Flow results revealed a considerable spread. Data improved after phantom correction. Both 4D and 2D Flow MRI revealed concordant results to differentiate patients from healthy individuals, especially based on values derived from anatomical parameters. The visualization of a vortex, indicating the presence of PH was achieved in 9 /11 patients and 2/35 volunteers.

**Discussion:**

This study confirms that quantitative parameters used for characterizing pulmonary hypertension can be gathered using 4D Flow MRI within clinically reasonable limits of agreement. Despite its unfavorable spatial and lesser temporal resolution and a non-neglible spread of results, the identification of diseased study participants was possible.

## Introduction

Pulmonary hypertension (PH) is a severe and multifactorial disease associated with a high mortality that can further aggravate the underlying disease. PH usually presents with non-specific symptoms and is thus often diagnosed at a late stage, thus impairing prognosis. Despite constant improvements of treatment, PH is still associated with a high mortality [1]. The diagnosis of PH is typically established by measuring an elevated mean pulmonary artery pressure (mPAP) of ≥ 25 mmHg at rest using invasive right heart catheterization (RHC) [2]. Although potential side effects of RHC are relatively rare, the invasive nature of the procedure bears a certain risk, especially in cardiopulmonary diseased patients, and may further delay diagnosis of PH. Moreover the clinical standard for diagnosing and characterizing PH still comprises a large set of additional exams, including computed tomography and ventilation/perfusion scintigraphy for diagnosis and subclassification of PH [2]. Therefore, a comprehensive, non-invasive diagnostic approach that is able to diagnose PH and potentially contribute further information regarding anatomy and hemodynamics would be of high relevance. Here, 4D Flow MRI may serve as a valuable tool in a comprehensive MRI setup.

There is a growing body of MRI studies evaluating PH, including cardiac magnetic resonance (CMR) [3–5], MRI of the lung parenchyma and its perfusion [6], and derived parameters such as transit time measurements [7]. Several studies tested the use of time resolved 2D phase contrast flow measurements, and more recently, 4D phase contrast magnetic resonance imaging (2D Flow MRI and 4D Flow MRI, respectively) for diagnosing PH. Among others, Sanz and co-workers successfully evaluated anatomical and hemodynamic parameters of the main as well as the proximal right and left pulmonary arteries based on 2D Flow MRI. Minimal vessel area, average blood flow velocity, and strain, defined as the relative vessel area change through the cardiac cycle, demonstrated a good sensitivity and specificity diagnosing PH [8, 9]. In addition, the mPAP has been successfully estimated based on 2D Flow MRI by Swift and co-workers [10].

4D Flow MRI has provided unprecedented insights into hemodynamics of various diseases and vascular territories [11]. In pulmonary hypertension, Reiter and co-workers established means of non-invasively diagnosing pulmonary hypertension by estimating the mPAP using presence and persistence time of secondary flow patterns in the main pulmonary artery [12–14]. Other data revealed differences in hemodynamic parameters derived from 4D Flow MRI between patients and volunteers [15].

The aforementioned MRI studies typically used a single technique to examine volunteers and patients. In the clinical setting, it is of equal interest how different diagnostic approaches compare. In addition, there is still concern regarding the errors associated with phase contrast acquisitions, the accuracy of the used sequences, and the possible impairment of phase contrast data by phase offsets [16]. Therefore, studies evaluating both the diagnostic performance and the comparability of established parameters are of practical interest. To the best of our knowledge, there is no comprehensive study comparing 4D Flow MRI to a broad set of MRI-based alternative measurements in patients with PH in vivo. Prior to its introduction into clinical routine, a thorough comparison and error quantification is essential.

Therefore, the goals of this study were: i) to compare 4D and 2D Flow MRI for differentiating between patients with PH and healthy volunteers using hemodynamic and anatomical parameters previously established for diagnosing PH; ii) to compare results from the applied 4D Flow MRI sequence on a digital broadband 3T MR system to those from established MRI sequences (2D Flow MRI and right ventricular volumetry, respectively), by performing phantom correction and conservation of mass (COM)-analysis and iii) to analyze the additional diagnostic value of secondary flow patterns in 4D Flow MRI.

## Materials and methods

### Study participants

All human subjects were enrolled in this HIPAA-compliant study after approval of the local ethics committee (Ethics Committee of the University of Lübeck, Study-ID: Az 13–117) and written informed consent. Forty-six subjects, 11 patients with clinically proven PH [PAT], 15 age-matched, healthy volunteers [VOL-O], and 20 young, healthy volunteers [VOL-Y] were included. Table 1 provides a full demographic description of study participants. According to the updated clinical classification by Simonneau et al., three patients presented with PH type I, four with PH type III, three with type IV and one patient with type V [2, 17]. Patients were classified following a diagnostic work-up in line with recent guidelines, including a qualifying right heart catheterization with an mPAP ≥25 mmHg. Patients presented with a mean mPAP of 46.1 ± 16.0 mmHg, mean systolic pulmonary artery pressure of 67.3 ± 11.1 and mean diastolic pulmonary artery pressure of 9.6 ± 4.2 mmHg according to RHC. The mean time interval between RHC and MRI was 12.5 ± 15 days. Exclusion criteria were: generally accepted contraindications against conducting MRI; inability to or withdrawal of consent into the study, and arrhythmia preventing completion of the ECG-gated studies.

**Table 1:** Demographics and physiological data.

	Total	VOL-Y	VOL-O	PAT	P value^#^
n	46	20	15	11	-
Age	44.1 ± 20.4	23.4 ± 2.2	56 ± 11.3	62.5 ± 16.3	0.24
Gender ratio [m:f]	20:26	7:13	6:9	7:4	0.23
Height [cm]	173 ± 10.6	176 ± 10.6	172 ± 11.7	168 ± 7.8	0.62
Weight [kg]	68.9 ± 16.2	64.5 ± 13.2	70.6 ± 17.9	75.0 ± 18.2	0.53
BMI [kg/m^2^]	22.8 ± 3.8	20.5 ± 2.0	23.5 ± 3.6	25.7 ± 4.3	0.19
Blood pressure [mmHg]	130±17/ 82±9	118±11/ 78±6	128±19/ 82±13	144±23/ 84±10	0.05*/0.72
Heart rate [bpm]	65.2 ± 11.0	62.3 ± 9.9	64.0 ± 10.6	70.0 ± 12.2	0.38

Table 1—Demographics and physiological data of study participants. Values are given as mean ± standard deviation

^#^ comparison between age-matched healthy volunteers and patients; asterisk (*) indicates statistical significance.

### MRI scans

MR Imaging was performed on a 3T MR scanner (Philips Ingenia Omega dStream, R5.18, Philips, Best, The Netherlands) using a 20-channel body surface coil. The digitization of the MR data took place in the coil itself rather than prior conversion to direct current, which, according to the manufacturer, is beneficial in terms of noise reduction. In all subjects, 4D and 2D Flow MRI were performed. For the secondary study goal, 23 healthy volunteers underwent RV cardiac volumetry and phantom scans repeating the 2D and 4D Flow MRI sequences in an identical manner on a static phantom were conducted based on the method proposed by Chernobelsky et al. [18].

As part of a clinically indicated cardiac MR exam protocol, patients were given 0.1ml/kg body weight contrast agent (Gadubotrol, Gadovist^®^, Bayer Vital, Leverkusen, Germany) at 1,5ml/sec using an Accutron MR^®^ injector (MedTron AG, Saarbrücken, Germany) in sequences conducted prior to the flow acquisitions. Volunteers did not receive contrast agent.

### 2D Flow MRI

One-directionally (“through-plane”) velocity encoded, referenced 2D phase contrast MRI sequences with retrospective ECG-gating were adapted to scanning within a single breathhold ≤ 20 seconds. A total of three 2D acquisition planes were carefully positioned perpendicular to the main pulmonary artery (MPA) approximately 1 cm downstream the pulmonary valve and in the left and right pulmonary artery (LPA, RPA, respectively) approximately 1 cm downstream the pulmonary bifurcation. The total scan time for all three 2D Flow MRI sequences ranged between 7:39 and 10:21 minutes. Typical imaging parameters were: repetition time/echo time (TR/TE) = 3.12/1.14ms; flip angle = 7°; velocity encoding sensitivity (V_enc_) = 80-120cm/s and an acquired in-plane resolution of 2.0 x 2.0mm and a slice thickness of 7mm. All scans were checked for velocity-aliasing directly following the end of each scan and repeated with adapted V_enc_-settings if necessary. Data were reconstructed to 40 temporal frames resulting in an effective temporal resolution of 17-34ms.

### 4D Flow MRI

For 4D Flow MRI acquisitions, a retrospectively ECG-gated, time-resolved, three-dimensional, cartesian phase-contrast MR sequence with referenced three-directional velocity-encoding was used. Respiratory gating with an acceptance window of 8-12mm to achieve a gating efficiency on the order of 60% was applied. To avoid velocity aliasing, the V_enc_ was adapted to the V_enc_ setting used in the previously obtained 2D Flow MRI sequences, which were checked for velocity aliasing. Typical imaging parameters were: 25–36 slices in oblique axial orientation; field of view = 180-300mm x 172-300mm x 167-294mm adapted to the individual anatomy; an acquired in-plane isotropic voxel resolution of 2.0–2.4mm was reconstructed to 2mm; TR/TE = 3.12/1.14ms; flip angle = 14°. To compensate potential signal-to-noise ratio differences between contrast enhanced and non-contrast enhanced scans, the flip angle was adapted to 7° in studies without contrast agent [19]. Data were reconstructed to 20 frames resulting in an effective temporal resolution of 36-67ms depending on the average heart rate. The use of parallel imaging with a SENSE factor of 2 in AP-direction allowed for effective scan times between 7:55 and 14:30 minutes, depending on the ECG trigger and navigator gating efficiency. If contrast agent was administered, the 4D Flow MRI sequence was obtained directly after a clinically requested contrast-enhanced MR angiogram to take advantage of the improved signal-to-noise and velocity-to-noise ratio [20]. Eddy currents and Maxwell terms were corrected automatically during offline reconstruction of the raw data.

### Right heart volumetry

A time-resolved, retrospectively ECG-triggered, balanced steady-state free precession (bSSFP) sequence planned transversal to the body axis covering the entire right ventricle was acquired per clinical routine during multiple breathholds for quantification of right ventricular SV [21]. Scan time for the bSSFP sequenced ranged between 3:21 and 4:46 minutes. Imaging parameters included: TR/TE = 3.12/1.14ms; flip angle = 45°; field of view = 390mm × 390mm; slice thickness = 8mm, interpolated to a spatial resolution of 1.5 × 1.5 × 8mm. Images were reconstructed to 40 cardiac frames through the cardiac cycle.

### Data analysis

Data were transferred to an offline workstation for postprocessing, visualization and quantification. The evaluation scheme for 4D Flow MRI on a workstation equipped with GTFlow (v2.2.15, GyroTools, Switzerland) included noise filtering of data, creation of a surface shaded 3D volume display based on the velocity weighted magnitude data (complex difference), and placement of cutplanes transecting the main, proximal left and right pulmonary arteries for further analysis.

**Fig 1:**
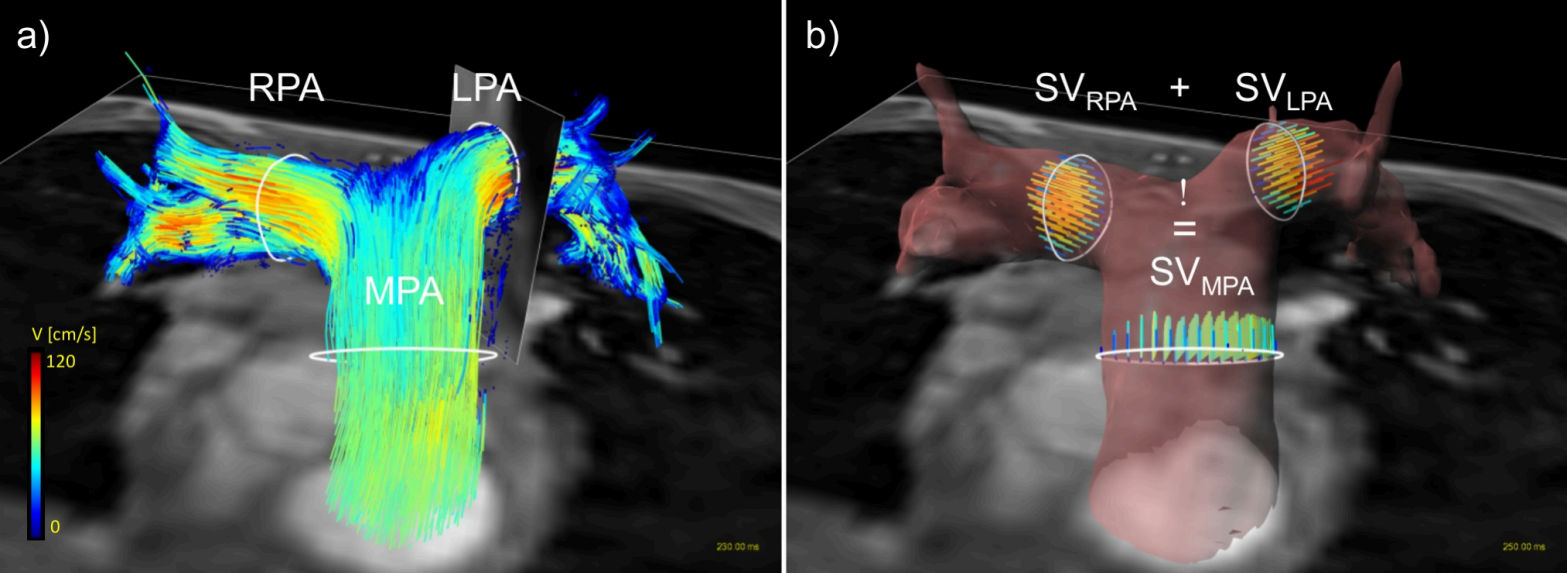
Data analysis of a 4D Flow MRI dataset. Fig 1 a) shows contour-placement (white) in the main, right and left pulmonary artery (MPA, RPA, LPA, respectively) in a healthy volunteer. The oblique cutplane transecting the LPA matches the 2D Flow geometric orientation to guarantee the exact comparability of data. Visualization in (a) was achieved by using particle-traces, projected in both flow directions, with contours as seed points. Color-coding was performed with respect to the acquired velocities. (b) the principle of the Conservation of mass-analysis in the same volunteer. Stroke volume in the MPA should equal the sum of stroke volume in the RPA and LPA to proof data consistency. The vessel wall was reconstructed based on complex difference and surface shading of the 4D Flow MRI data.

For quantitative analyses, the anatomical position of the 2D Flow MRI slices was automatically extracted from the DICOM data in order to define exactly matching cut-planes in the 4D Flow MRI data volume. Correct positioning of these planes was confirmed via a 3D angiogram, computed from phase-difference information derived from the 4D Flow dataset. On each vessel cutplane, vessel walls were segmented with a B-spline interpolation algorithm. Manual segmentation of the vessel wall was performed as described before with established small inter- and intra-reader differences [22]. The segmentation was copied over all time frames and was then carefully adapted manually to each time point through the cardiac cycle accounting for pulsation and vessel motion. An example of cutplane placement, contouring, and subsequent visualization can be found in Fig 1A. Extracted hemodynamic and anatomical parameters from 2D and 4D Flow MRI used for comparison were: Stroke volume (SV), maximum flow (Q_max_), timepoint of maximum flow (t_Q_max_), maximum velocity (v_max_), timepoint of maximum velocity (t_v_max_), maximum area (A_max_) and minimum area (A_min_). Strain was calculated as (A_max_-A_min_)/A_min_*100 [9]. In addition, a COM-analysis following the logic that flows in RPA and LPA must add up to flow in the MPA (SV_MPA_ = SV_LPA_ + SV_RPA_) as further illustrated in Fig 1B [23] was performed. For error quantification, the geometric and anatomical information of the above MPA, LPA and RPA cutplanes of the 2D and 4D data were copied to the phantom data to achieve perfect spatiotemporal matching.

The left ventricular stroke volume (SV) based on bSSFP CMR was determined per clinical standard according to the Simpson’s method using the Extended MR WorkSpace (v2.6.3.5, Philips) by segmenting endocardial borders on end-diastolic and end-systolic frames on every slice through the right ventricle. Resulting end-diastolic and end-systolic volumes were subtracted to calculate SV.

For blood flow pattern visualization, time-resolved pathlines emitted up- and downstream from each predefined contour and instantaneous 3D streamlines were used on the 4D Flow MRI data. Both visualization options were based on the interpolated spatial resolution of 2mm isotropic voxel size. Data were color-coded with respect to the acquired blood flow velocity. For details on the visualization options please see [20]. Both visualization strategies were taken into account for the evaluation of secondary vortices. The presence of secondary vortices defined as concentric circular flow patterns within the vessel was recorded [24].

### Statistical analysis

Statistical analyses were performed using XLSTAT^®^ (Addinsoft^®^, New York, United States). All continuous values are presented as average ± standard deviation. The 95% confidence interval was calculated for each parameter. All continuous data were tested for normal distribution using Shapiro-Wilk test.

After normal distribution was confirmed, statistical testing for significant differences was achieved using a two-sided unpaired Student’s t-test between patients with PH and age-matched, healthy volunteers. Significance was accepted at a p-value of <0.05. Sensitivity and specificity based on cut-off values for the detection of PH proposed by Sanz and colleagues for A_min_ (660mm2) [9] and Strain (24%) [8] were calculated. Receiver operating characteristic (ROC) curve analysis and area under the curve calculation were performed for both parameters.

To evaluate quantitative parameters between 4D, 2D Flow MRI, and right heart volumetry a Bland-Altman analysis was performed accepting differences in the range of ± 10% between tests as clinically acceptable. Bland-Altman data and plots are given as mean bias and limits of agreement (± 1.96 standard deviations of the difference) [25]. Scatter plots of 4D Flow versus 2D Flow MRI were constructed to depict correlation. In addition the intraclass correlation coefficient (ICC; two way mixed-effects model; absolute agreement) was computed to test for agreement between 4D and 2D Flow MRI, respectively. ICC values below 0.50 were evaluated as poor, between 0.50 and 0.75 as moderate, between 0.75 and 0.90 as good and values greater than 0.90 as excellent reliability [26].

For the analysis of additional value of 4D Flow MRI, sensitivity and specificity for the occurrence of secondary vortices in the MPA to detect subjects presenting with an mPAP of ≥ 25mmHg confirmed by RHC-examination were computed.

## Results

### Comparison between volunteers and patients with PH

Table 2 provides a comparative overview of results in patients with pulmonary hypertension and age-matched volunteers. 4D and 2D Flow MRI both revealed the ability to distinguish patients from healthy individuals. Overall, patients with pulmonary hypertension presented with increased vessel areas, decreased flows and flow velocities and their respective times to maximum, as well as decreased strain. Differences of the anatomical parameters A_max_, A_min_, and strain revealed statistical significance for both, 4D and 2D Flow MRI.

**Table 2:** Comparison of hemodynamic and anatomical parameters between aged-matched, healthy volunteers (VOL-O) and patients with PH (PAT).

	4D Flow MRI		P value	2D Flow MRI		P value
	VOL-O	PAT		VOL-O	PAT	
SV [ml]	81.1 ± 12.8	79.3 ± 17.5	0.8	82.0 ± 16.0	70.7 ± 17.1	0.1
**Q**_**max**_ **[ml/s]**	334.1 ± 66.0	321.3 ± 83.7	0.7	363.3 ± 73.3	356.2 ± 85.7	0.8
**t_Q**_**max**_ **[ms]**	136 ± 23.9	114.8 ± 21.5	<0.05*	124,7 ± 25.3	104.5 ± 28.5	0.07
**v**_**max**_ **[cm/s]**	87.9 ± 16.5	83.3 ± 17.6	0.5	96.8 ± 18.5	80.3 ± 15.9	<0.05*
**t_v**_**max**_ **[ms]**	143.2 ± 40.5	114.8 ± 21.5	0.2	142.18 ± 50.5	131.47 ± 29.7	0.5
**A**_**max**_ **[mm2]**	730.2 ± 140.7	1152.9 ± 237.4	<0.01*	666.2 ± 114.0	1071.1 ± 279.6	<0.01*
**A**_**min**_ **[mm2]**	545.7 ± 106.7	927.5 ± 118.2	<0.01*	473.8 ± 95.5	906.5 ± 204.7	<0.01*
**Strain [%]**	34.8 ± 13.3	24.5 ± 8.8	<0.05*	41.2 ± 12.4	21.2 ± 7.3	<0.01*

Values are given as average ± standard deviation; asterisk (*) indicates statistical significance.

**Fig 2:**
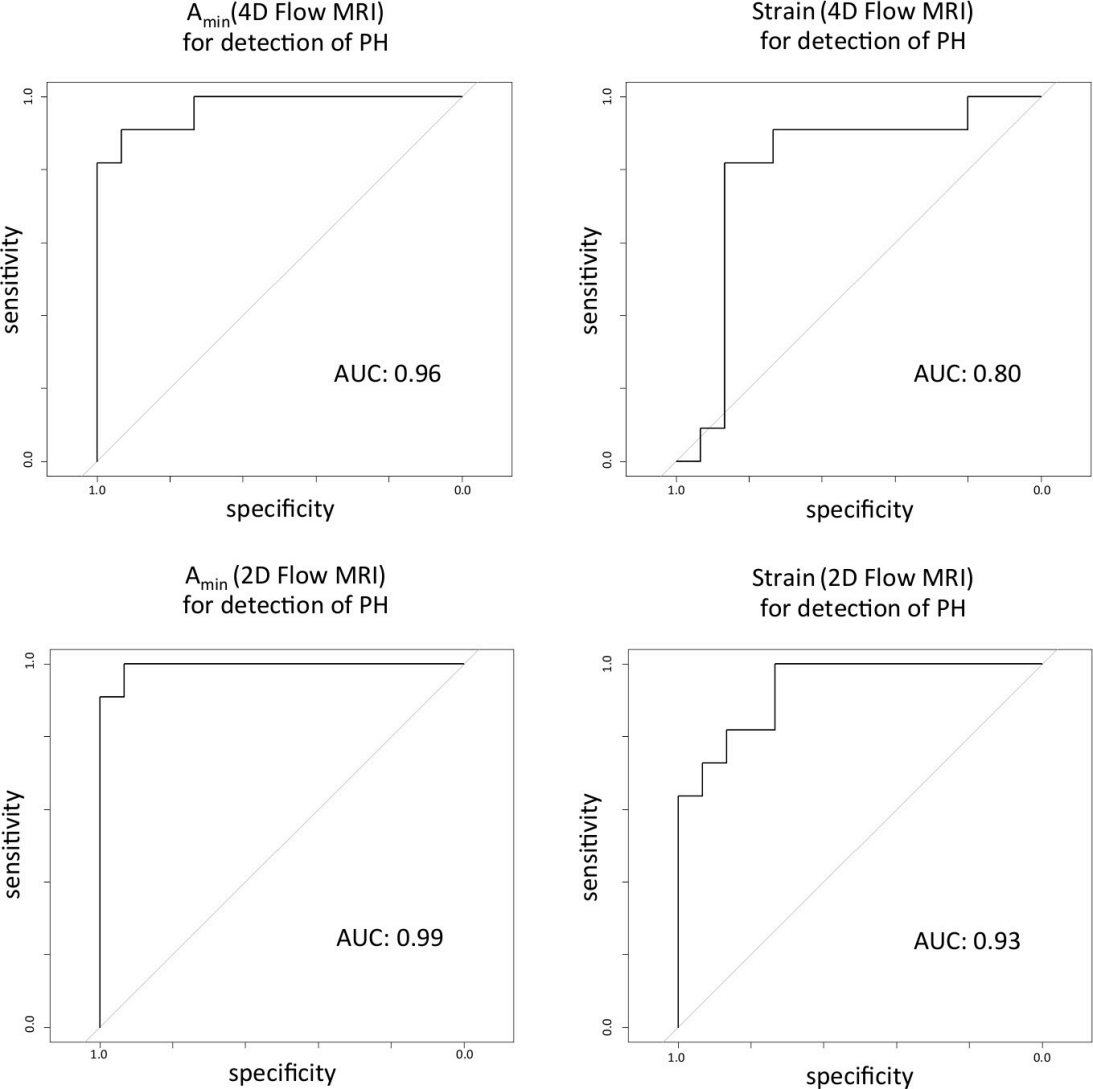
Receiver operating characteristic curves for A_min_ and Strain measured with 4D and 2D Flow MRI. The ROC curves illustrate the ability of A_min_ and Strain to detect patients with PH, characterized by a mean pulmonary artery pressure of > 25 mmHg. AUC = area under the curve.

Applying the aforementioned cut-off values introduced by Sanz et al. yielded the following results for the differentiation between healthy and diseased subjects: A_min_ reached a sensitivity of 90.9% and specificity of 93.3% for detecting PH for both techniques. Values for strain were especially helpful for diagnosing PH taking into accounts its high specificity of 86.7%/100% for both 4D and 2D Flow MRI, respectively. The sensitivity to detect PH by MRI based on strain analysis in the PA revealed values of 54%/63% for 4D and 2D Flow MRI, respectively. ROC curves (Fig 2) confirmed the excellent ability of A_min_ to detect patients with PH for both techniques, with 2D Flow MRI being slightly superior to 4D Flow MRI. Despite a large difference in means for v_max_ for both techniques, differences between patients and age-matched healthy volunteers showed statistical significance for the 2D Flow MRI acquisitions only (p<0.05). For the time to maximum flow (t_Q_max_) differences reached statistical significance only for 4D Flow acquisitions (p<0.05).

### Comparison of hemodynamic and anatomical parameters

Table 3 summarizes overall hemodynamic and anatomical results without phantom correction, averaged over the group of 20 young, 15 age-matched healthy volunteers and 11 patients. There was good agreement between 2D and 4D Flow MRI with a tendency of 4D Flow MRI to overestimate results in comparison to 2D Flow MRI. Only Q_max_ (4D: 359.6 ± 92.2ml; 2D: 369.7 ± 82.3ml, p = 0.1) presented with a tendency towards lower values in 4D than in 2D Flow MRI.

**Table 3:** Comparison of hemodynamic and anatomical values between 2D and 4D Flow MRI in the entire study collective.

	4D Flow MRI	95% CI	2D Flow MRI	95% CI	P value
**SV [ml]**	88.6 ± 21.3	[82.4; 94.7]	82.8 ± 18.9	[77.3; 88.3]	< 0.01*
**Q**_**max**_ **[ml/s]**	359.6 ± 92.2	[332.8; 386.6]	369.7 ± 82.3	[341.9; 391.0]	0.1
**t_Q**_**max**_ **[ms]**	136 ± 23.9	[131.7; 150.5]	136 ± 23.9	[122.0; 138.7]	< 0.05*
**v**_**max**_ **[cm/s]**	91.1 ± 19.5	[96.8; 85.5]	87.4 ± 17.2	[82.5; 92.4]	0.2
**t_v**_**max**_ **[ms]**	147.5 ± 56.9	[131.1; 164.0]	142.18 ± 50.5	[125.5; 149.3]	0.3
**A**_**max**_ **[mm**^**2**^**]**	865.0 ± 237.4	[797.2; 934.5]	789.2 ± 233.5	[721.7; 856.7]	0.1
**A**_**min**_ **[mm**^**2**^**]**	626.5 ± 209.5	[566.0; 687.1]	565.6 ± 228.8	[499.5; 631.7]	0.2
**Strain [%]**	41.3 ± 18.5	[36.0; 46.6]	46.1 ± 20.9	[40.1; 52.2]	0.2

Values are given as average ± standard deviation; asterisk (*) indicates significance.

**Fig 3:**
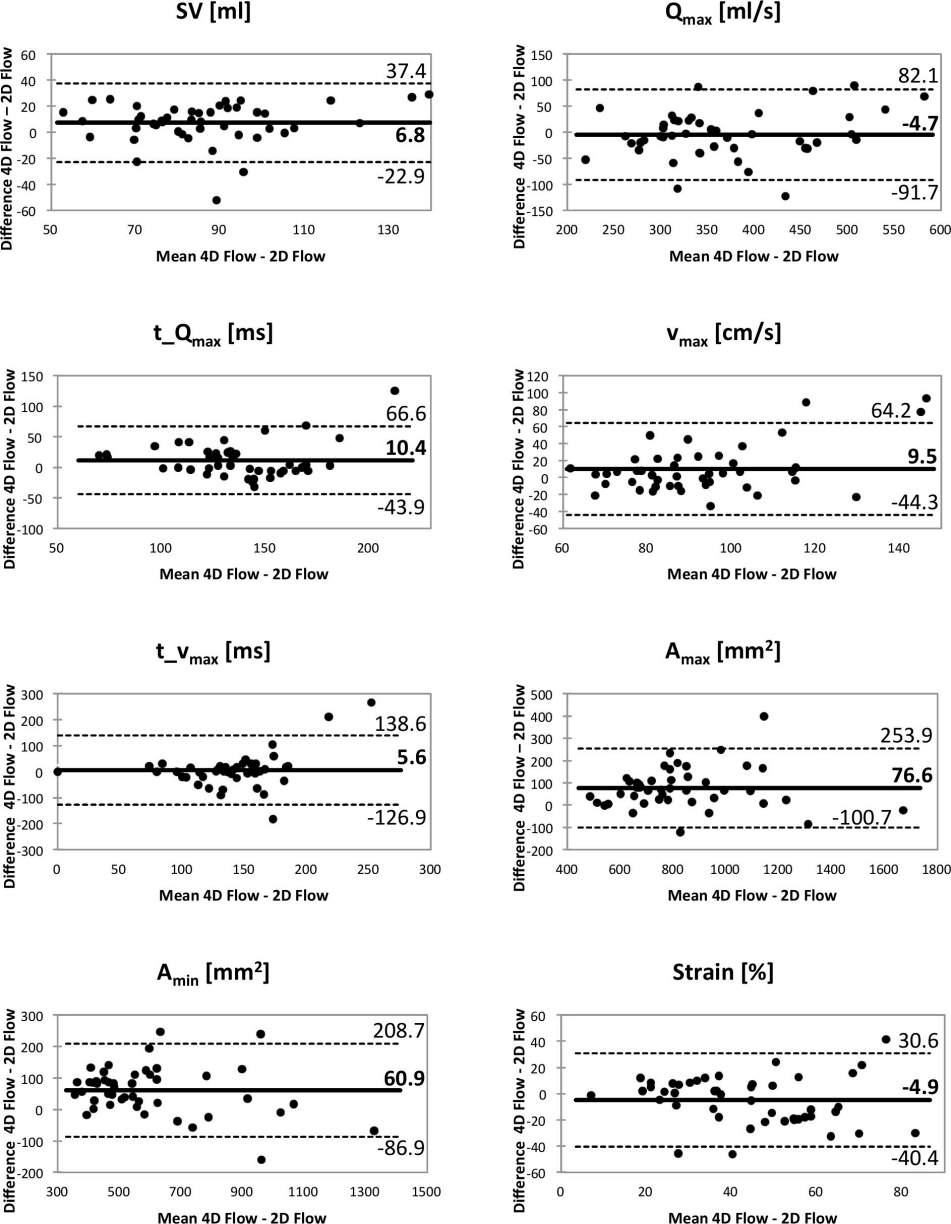
Bland-Altman plots for all evaluated hemodynamic and anatomical parameters for the comparison between 4D Flow and 2D Flow MRI. The solid line indicates mean bias between techniques, dashed lines indicate limits of agreement (mean bias ± 1.96 standard deviations).

**Fig 4:**
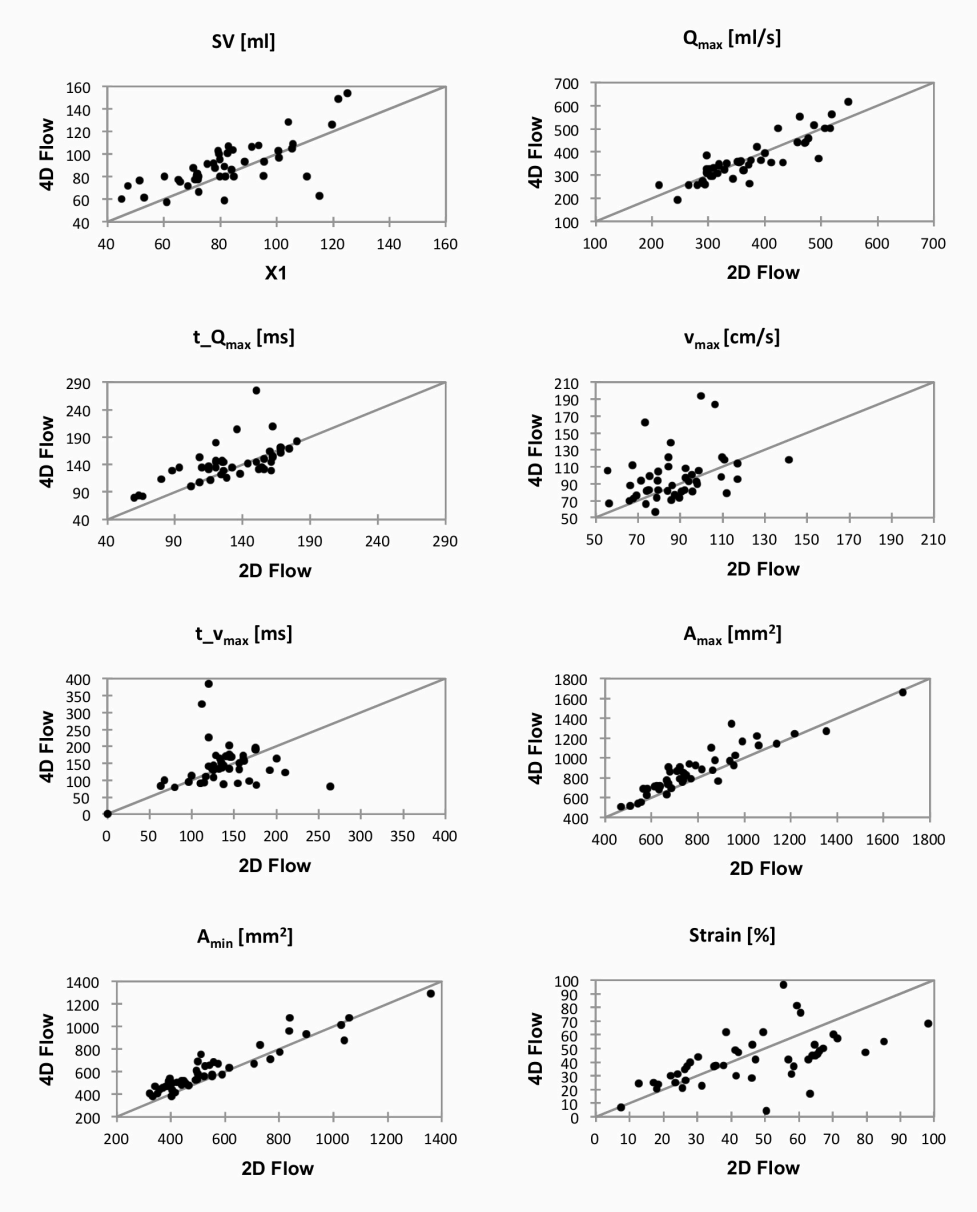
Scatter plots for all evaluated hemodynamic and anatomical parameters for the comparison between 4D Flow and 2D Flow MRI.

Overall Bland-Altman analysis and the ICC confirmed these findings. While the mean error was below 10% for all parameters except for A_min_ (60.9 ± 75.4 mm^2^; 12% ± 12%), in particular the parameters t_v_max_ (5.6 ± 129.9cm/s; 7% ± 45%) and strain (-4.9 ± 35.5%; -9% ± 43%) revealed considerable spread. ICC demonstrated good to excellent reliability for all quantitative parameters with the exception of t_Q_max_ and t_v_max_. Bland-Altman plots are displayed in Fig 3, corresponding scatter plots can be found in Fig 4, and ICC values are given in Table 4.

**Table 4:** Intraclass correlation coefficients with corresponding 95% confidence intervals for quantitative parameters evaluated with 2D and 4D Flow MRI in the entire study collective.

	ICC	95% CI
**SV [ml]**	0.82	[0.66; 0.91]
**Q**_**max**_ **[ml/s]**	0.93	[0.88; 0.96]
**t_Q**_**max**_ **[ms]**	0.73	[0.50; 0.85]
**v**_**max**_ **[cm/s]**	0.82	[0.67; 0.90]
**t_v**_**max**_ **[ms]**	0.19	[-0.46; 0.55]
**A**_**max**_ **[mm2]**	0.94	[0.70; 0.98]
**A**_**min**_ **[mm2]**	0.95	[0.78; 0.98]
**Strain [%]**	0.78	[0.60; 0.88]

### Phantom corrected comparison

The comparison of SV in 23 volunteers examined with all three imaging techniques revealed good agreement: SV obtained in the MPA was 93.0 ± 26.1 ml for 4D Flow MRI and 87.3 ± 19.1 ml for 2D Flow MRI. The mean bias of 5.7 ± 10.6 ml revealed by the Bland-Altman Plot was acceptably small. The average SV derived from CINE-bSSFP was 90.5 ± 25.3 ml, resulting in a mean bias compared to 4D Flow MRI of 5.5 ± 17.2 ml. Phantom correction further improved mean bias to 3.1 ± 12.3 ml (4D Flow vs. 2D Flow MRI) and to -0.1 ± 20ml (4D Flow MRI vs. CINE-bSSFP), decreasing the Bland-Altman window width, too.

Similar to volumes, the peak velocity revealed good agreement between 2D Flow and 4D Flow MRI of 89.4 ± 14.9 cm/s and 93.3 ± 16.3 cm/s. Bland Altman analysis demonstrated a mean bias of 3.9 ± 12.5 cm/s which was further improved by phantom correction to 1.3 ± 14.5 cm/s.

The COM-analysis for the uncorrected 4D Flow MRI data in 23 volunteers presented matching average results of 93.0 ± 26.1ml for SV_MPA_ and 90.8 ± 19.5ml for the sum of SV_RPA_ + SV_LPA_ (p = 0.51). Mean bias did not benefit from phantom correction revealing an increase in difference from 2.2 ± 15.6 ml (uncorrected data) to 7.7 ± 15.9 ml (corrected data).

### Visualization of secondary vortices

**Fig 5:**
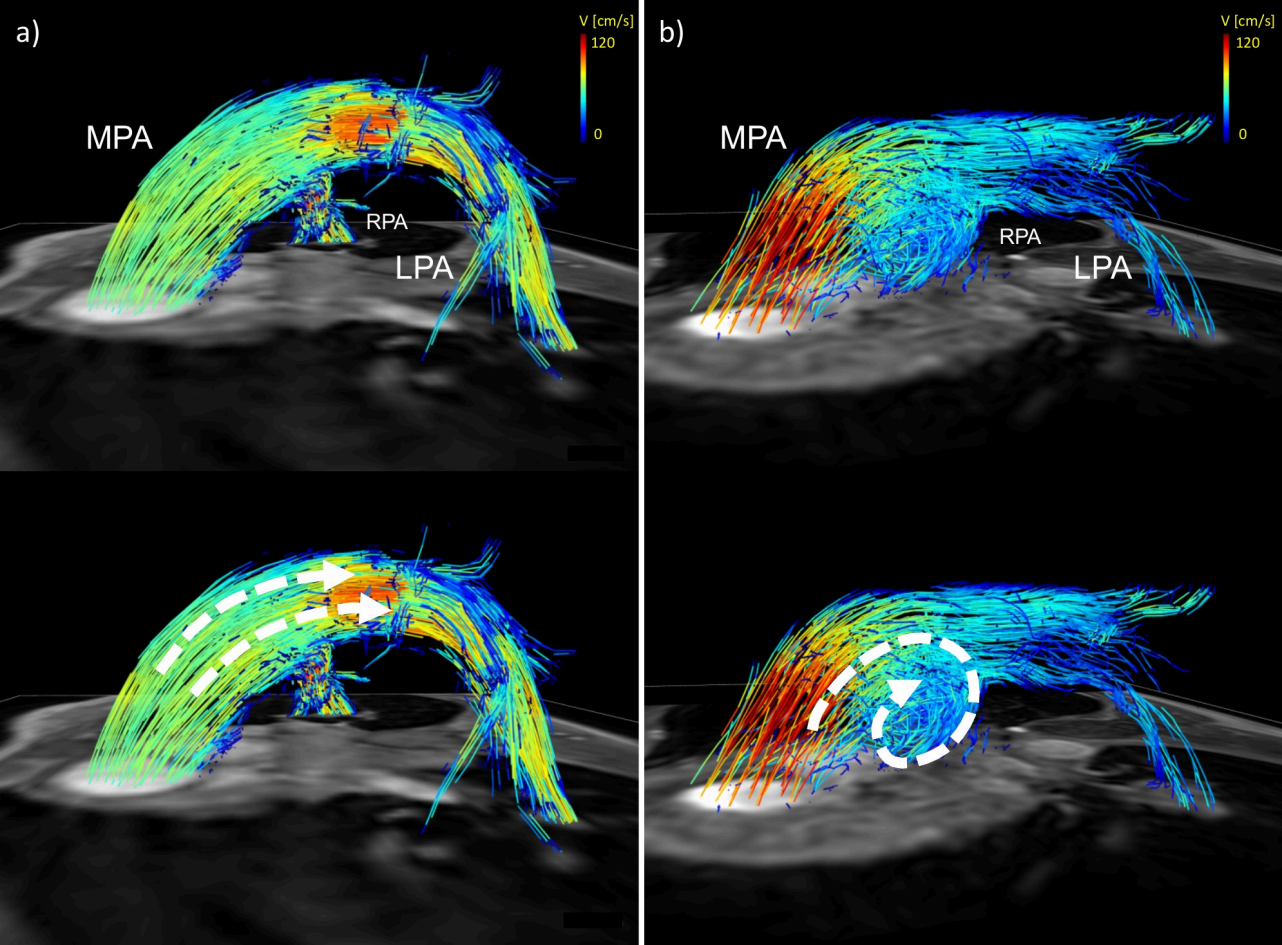
Visualization of macroscopic bloodflow in a healthy volunteer and a patient with PH. The volunteer (a) shows physiological laminar flow without any secondary flow patterns whereas the patient (b) demonstrates a vortex in the MPA, indicating an increased mean pulmonary artery pressure.

The flow pattern visualization revealed secondary vortices in the distal MPA in 9 of 11 patients and in 2 of 35 volunteers, resulting in a sensitivity of 81.8% and a specificity of 94.6% for detection of PH in this study. An example of blood flow visualization can be found in Fig 5.

## Discussion

The herein presented data are unique in that they express an ambiguity that seems to be inherent to the field of 4D Flow MR imaging: To one end, 4D Flow MRI is able to aid the detection of PH and distinguish PH patients from volunteers based on hemodynamic and anatomical parameters as well as flow visualization. To the other end, while showing a convincing degree of comparability between clinically applicable MR methods, there is still a considerable spread of differences and a tendency of 4D Flow MRI to overestimate certain parameters. This has to be taken into account when evaluating patients’ quantitative data. However, the advantage of the 4D Flow MRI approach is that a single sequence provides both anatomical and hemodynamic values as well as bloodflow visualization guiding the detection of PH while a plethora of further derived parameters potentially being available [11, 23].

Despite the restrictions mentioned above, results of our study suggest that 4D Flow MRI offers a feasible, comprehensive approach to evaluate quantitative parameters in the pulmonary circulation as underlined by the good to excellent reliability of flow, velocity and anatomical parameters in the ICC analysis. Cut off-values for detecting an elevated mPAP established previously for A_min_ and strain with 2D Flow MRI [8, 9] were confirmed in this study and successfully applied to data derived from 4D Flow measurements with comparable results. Sanz and colleagues reported sensitivities and specificities of 92%/88.2% for A_min_ [9] and 77%/95% for strain [8], matching the results of our study. While results for sensitivity and specificity regarding strain and A_min_ are favorable, the large spread for strain in particular revealed by BA-analysis potentially hampers the applicability of this parameter for diagnosing PH.

However, with a growing body of studies comparing 4D and 2D Flow MRI, both over- and underestimation of hemodynamic parameters measured with 4D Flow MRI have been reported for different vendors and systems in literature [22, 27–32]. Various reasons have been attributed to these differences; including general limitations inherent in 4D Flow MRI like decreased spatiotemporal resolution [20], as well as different acquisition and post-processing techniques [16]. While the decreased spatiotemporal resolution is likely responsible for the low reliability of t_Q_max_ and t_v_max_ demonstrated in the ICC analysis, these differences are contrasted by good results of the COM-analysis, suggesting reliable intrinsic consistency of our 4D Flow measurements [23]. This consistency of 4D Flow MRI implies that above differences in flow, velocity and anatomical parameters may have also been influenced by varying physiological conditions, especially image acquisition during free breathing in 4D Flow MRI as opposed to breathholding in 2D Flow MRI and bSSFP CMR [28, 33]. Similarly, there may have been bias introduced by the fact that 4D Flow MRI was performed after the application of contrast agent whereas 2D Flow MRI was performed before.

As expected, phantom correction improved comparability which may be due to imperfectly corrected eddy currents and the relatively high signal-to-noise ratio in 4D Flow MRI as compared to 2D Flow MRI [34]. A debatable finding of our study is the deterioration of the COM-analysis after phantom correction. While there is no simple, provable explanation to this finding, additional factors such as differing gradient temperatures during human and phantom studies may affect phase contrast measurements to a variable extent. Such variations are difficult to control, especially during the 4D Flow measurements with durations of over 10 minutes [34, 35]. As employing a phantom potentially doubles scan time and differences between non-phantom and phantom corrected data were relatively low, phantom correction might be dispensable for practical clinical use. However, the residual error and spread in both, corrected and non-corrected data, is still of note and should be motivation for future optimization of the sequence, the acquisition set-up, and the use of a phantom in quantitative studies.

In general, 4D Flow MRI offers advantages over 2D Flow MRI, such as the post-hoc analysis of the 4D volume that allows for targeted analysis of vessel sections of interest and the placement of multiple analysis planes, thus relieves one of the need to plan multiple slices during the acquisition and minimizes potential errors due to improper slice-placement limiting the use of 2D phase contrast MRI sequences. As demonstrated by various works including this study, pathological flow conditions may vary considerably in patients with PH. Placement of a single 2D slice in a complex flow field to analyze quantitative parameters may miss relevant information, whereas in a 4D Flow dataset analysis planes can be tailored to the individual patients flow characteristics. Technical limitations such as long scan times and the somewhat lengthy evaluation are subject of continuous improvements, including faster imaging protocols like compressed-sensing and novel software approaches [36, 37].

The peerless characteristic of 4D Flow MRI is the visualization of blood flow patterns in the pulmonary vasculature. We were able to demonstrate the presence of a vortex in the main pulmonary artery in our data as previously described [12], confirming good sensitivity and specificity in the detection of PH via visualization of secondary flow patterns. Other advanced parameters obtainable with 4D Flow MRI, such as vorticity and wall shear stress, show promising results in detecting PH und underscore the potential and comprehensive nature of this technique for clinical application [15, 38–40]. Unfortunately, the evaluation of these parameters was not possible with our software package, therefore warranting further research.

Other potential shortcomings of this study may be seen in the lack of a true reference standard (ground truth) for in vivo flow measurements. Additionally, certain comparison efforts were only performed in a subset of participants. Also including more patients is essential to future work elaborating on testing for PH and characterizing PH subtypes, in particular. The general limitations of 4D Flow MRI are well known and have been discussed extensively [20]. For further comparison interscan, inter-vendor as well as rescan or inter-field strength comparisons would be desirable. Possible, physiological diurnal variations of the acquired parameters have not been assessed to this date. Finally, the patients received a variability of different PH-treatments, which may have confounding effects on the study’s results.

## Conclusion

In conclusion, our study confirms both, the applicability of 4D Flow MRI applied on a digital broadband 3T MR system as well as acceptable agreement and reliability for most quantitative parameters in volunteers and patients with PH compared to 2D Flow MRI. Despite the considerable spread and potential overestimation in particular for strain and peak-time-related parameters that have to be taken into account when examining patients with 4D Flow MRI, 4D Flow MRI promises the most comprehensive evaluation of the pulmonary circulation provided by a single diagnostic technique, assessing hemodynamics, anatomy and secondary flow patterns non-invasively. To identify parameters suitable to better characterize and reliably diagnose PH using 4D Flow, larger, preferably multicenter studies with greater numbers of patients would be desirable.

## Supporting information

S1 File S1_Table1_geometrical_parameters. Quantitative values for geometrical parameters over all study participants for 4D and 2D Flow MRI.
(XLSX)

S2 File S2_Table2_Quanti_4D_2D_CMR_Phantom. Quantitative values for all study participants who received 4D, 2D Flow MRI, CMR and Phantom corrected measurements.
(XLSX)

S3 File S3_Table3_ 4D_2D_CMR_Phantom_COM/participants. Demographics and values for conservation-of-mass analysis for all study participants who received 4D, 2D Flow MRI, CMR and Phantom corrected measurements.
(XLSX)

S4 File S4_Table4_hemodynamical_parameters. Quantitative values for hemodynamic parameters over all study participants for 4D and 2D Flow MRI.
(XLSX)
